# Broadening the absorption bandwidth of metamaterial absorbers by transverse magnetic harmonics of 210 mode

**DOI:** 10.1038/srep21431

**Published:** 2016-02-18

**Authors:** Chang Long, Sheng Yin, Wei Wang, Wei Li, Jianfei Zhu, Jianguo Guan

**Affiliations:** 1 State Key Laboratory of Advanced Technology for Materials Synthesis and Processing, Wuhan University of Technology, Wuhan, 430070, China; 2 State Key Laboratory for Modern Optical Instrumentation, Centre for Optical and Electromagnetic Research, College of Optical Science and Engineering, Zhejiang University, Hangzhou 310058, China

## Abstract

By investigating a square-shaped metamaterial structure we discover that wave diffraction at diagonal corners of such a structure excites transverse magnetic harmonics of 210 mode (TM_210_ harmonics). Multi-layer overlapping and deliberately regulating period length between adjacent unit cells can significantly enhance TM_210_ harmonics, leading to a strong absorption waveband. On such a basis, a design strategy is proposed to achieve broadband, thin-thickness multi-layered metamaterial absorbers (MMAs). In this strategy big pyramidal arrays placed in the “white blanks” of a chessboard exhibit two isolated absorption bands due to their fundamental and TM_210_ harmonics, which are further connected by another absorption band from small pyramidal arrays in the “black blanks” of the chessboard. The as-designed MMA at a total thickness (*h*) of 4.36 mm shows an absorption of above 0.9 in the whole frequency range of 7–18 GHz, which is 38% broader with respect to previous design methods at the same *h*. This strategy provides an effective route to extend the absorption bandwidth of MMAs without increasing *h*.

Metamaterials possess exotic electromagnetic properties, and have important applications in diverse areas, such as sensors^1^,^2^,^3^, invisibility cloaks^4^,^5^,^6^, camouflage^7^,^8^ and energy harvesting^9^,^10^,^11^. Among them, metamaterial absorbers (MMAs) have attracted considerable attention from radio to optical frequency by virtue of the perfect absorption, thin thickness and flexible design ability of the metamaterials^9^,^10^,^11^,^12^,^13^,^14^. However, most of the microwave MMAs developed so far show a narrow absorption bandwidth, though many efforts including by optimizing artificial structure design^15^,^16^,^17^,^18^,^19^,^20^,^21^,^22^,^23^ have been made on the broadening of the absorption bandwidth of MMAs. In fact, the design of a broadband MMA is intractable due to the complicated interaction among different metamaterial resonant units, the limited room to place various metamaterial resonant units, as well as the intrinsic contradiction between the bandwidth and absorption strength by using too many kinds of metamaterial resonant units^24^. Thus, our research group has recently proposed a novel concept to skillfully integrate non-planar metamaterials with traditional magnetic absorbing materials and effectively manufactured absorbers with a significantly broadened absorption bandwidth in GHz frequencies. However, the dependency on the intrinsic dispersion and magnetic properties of the traditional magnetic absorber (TMA)^25^,^26^ restricts extension of the so-defined composite MMAs to other frequency ranges.

On the other hand, vertically assembling multiple layer metamaterials can merge their resonance absorption peaks from every layer of metamaterial structures, and consequently extend the absorption bandwidth of metamaterials without decreasing absorption strength^27^,^28^,^29^,^30^,^31^,^32^,^33^. But it generally has an disadvantage of a large thickness since the availability of a broad absorption bandwidth requires a number of layers. A multi-unit pattern method was used on the design of a pyramidal multi-layered MMA, reducing the MMA thickness by half in condition of the same absorption bandwidth^34^. Nevertheless, in all of these vertically assembling multiple layer metamaterials, every layer only contributes one narrow resonance peak to the absorption bandwidth^34^,^35^. Metal-dielectric-metal cavity structures can theoretically excite resonant absorption of multiple orders^36^,^37^,^38^,^39^,^40^, exhibiting multi-peak or multi-band absorption^41^,^42^,^43^. For example, a two different-sized hyperbolic metamaterial waveguide array has demonstrated a strong absorption in most of the frequency range between 2.3–40 GHz when the thickness is 11.52 mm^44^. However, the absorption band above 0.9 is separated and relative narrow. This is because the employed harmonic frequency adjacent to the fundamental harmonic one is twice larger than the latter, suggesting few possibility for these two kinds of resonance peaks to form a continuous high absorption band in limited thickness.

In this work, it is discovered that square-shaped metal-dielectric-metal cavities are able to excite weak TM_210_ harmonics, which are between the fundamental and third order harmonics. These weak harmonics can be remarkably enhanced by multi-layer overlapping into pyramidal arrays and deliberate regulation of period length between adjacent unit cells. The as-enhanced TM_210_ harmonic absorption can easily be connected with the fundamental harmonic absorption to form a singificantly broadened continuous strong absorption waveband by alternately inserting another pyramidal arrays of small sizes. The as-proposed multi-layered pyramidal MMAs have taken full use of both the fundamental and TM_210_ harmonics, and thus have a bandwidth of twice wider than those of the original simple counterparts. They have significant advantages of a highly absorption efficiency or a broad strong absorption bandwidth, even compared with the multi-unit MMAs reported previously^34^,^35^,^44^.

## Results and Discussion

**Figure 1:**
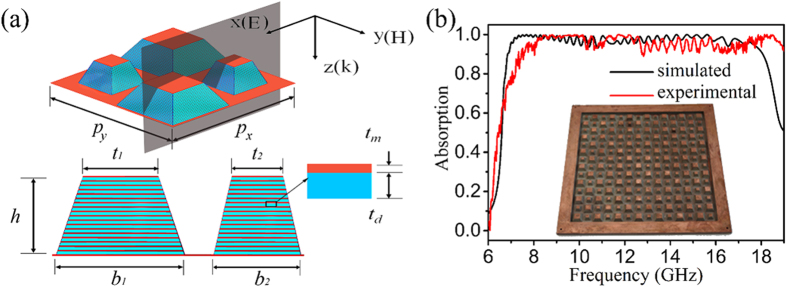
(**a**) 3-dimensional structure diagram (top) as well as a typical cross-section along *x-z* shadow plane (bottom) for the repeating unit of the as-designed metamaterial absorber (MMA) labelled with sizes. The red brown represents metal copper foils and the light blue represents dielectric FR-4. *p*_*x*_ = 24 mm, *p*_*y*_ = 24 mm, *t*_*1*_ = 6.3 mm, *t*_*2*_ = 3.6 mm, *b*_*1*_ = 11 mm, *b*_*2*_ = 6.6 mm, *t*_*m*_ = 0.018 mm, *t*_*d*_ = 0.2 mm, and *h* = 4.36 mm. (**b**) The simulated and experimental absorption spectra of the as-designed MMA. The inset shows a photograph of the fabricated MMA.

We achieve broadband, thin-thickness metamaterial absorbers (MMAs) by fully taking advantages of both the fundamental and TM_210_ harmonics. This is quite different from the recently reported two different-sized hyperbolic metamaterial waveguide array^44^, despite the apparently similar structure between them. In the latter hyperbolic metamaterial waveguide array structure, no high order harmonic is empolyed between the third order and fundamental harmonics, only resulting in a few of separated strong absorption bands in a wide frequency range. To illustrate the effectiveness of our designing strategy, we take a broadband multi-layered MMA in the range of 6–19 GHz for an example. Figure 1 depicts the structure and absorption spectra of the MMA. It consists of two different-sized pyramids, which are arranged in a chessboard manner. Each pyramid is composed of 20 layers of metal-dielectric-metal resonance cavities with their sizes tapered linearly from top to the bottom. Each thin layer of metal-dielectric- metal resonance cavity is made of a lossy dielectric material epoxy-based FR4 sandwiched between two copper foils. The copper foils have an electric conductivity of 5 × 10^7^ S/m while the FR4 has a relative permittivity of 4.3(1-0.025i). Figure 1(a) shows the schematics of the three-dimensional structure and a typical cross-section parallel to *x-z* plane for a unit cell of the MMA. In the simulations, periodic boundary conditions are used in the *x* and *y* directions, and a plane wave is incident downward on the MMA with the electric field polarized along the *x*-direction (TE wave, transverse electric) as indicated in Fig. 1(a). The designed dimensions of a unit cell shown in Fig. 1(a) are *p*_*x*_ = 24 mm, *p*_*y*_ = 24 mm, *t*_*1*_ = 6.3 mm, *t*_*2*_ = 3.6 mm, *b*_*1*_ = 11 mm, *b*_*2*_ = 6.6 mm, *t*_*m*_ = 0.018 mm, *t*_*d*_ = 0.2 mm, and *h* = 4.36 mm. As the MMA is backed with a copper foil, no transmission could be made and the absorption can be calculated by *A* = 1* − R*. Figure 1(b) shows the simulated and measured absorption spectra of the as-designed MMA under normal incidence in frequencies (*f*) of 6–19 GHz. The MMA shows a strong simulated and measured absorption of above 0.9 in a wide frequency range of more than 11 GHz. The good agreement between the experimental and simulated results indicate that our design method is viable. The calculated absorption bandwidth (7–18 GHz) has a small shift to a low frequency with respect to the measured one (8–19 GHz). This could be reasonably explained by the unavoidable but acceptable fabrication errors. The fluctuation in the simulated curve indicates that the broadband absorption spectrum resulted from the combination of many neighbour absorption peaks together, consistent with the previous finding for the metamaterials made of truncated pyramids^10^,^27^.

For our designing MMA, the relative absorption bandwidth (*W*_RAB_ = 2(*f*_U_ − *f*_L_) ⁄ (*f*_U_ + *f*_L_), where *f*_U_ and *f*_L_ are the upper and lower frequency bounds of a continuous band with absorption above 0.9) is calculated to be as large as 88%, surpass the planar microwave MMAs reported so far^27^,^28^,^41^,^44^. However, *W*_RAB_ cannot fully evaluate the absorption efficiency of MMAs as it does not take into account the MMA thickness. Here a ratio of operational bandwidth to thickness *W*_ob/*t*_ = (λ_U_ − λ_L_)/*t* is proposed, where λ_U_ and λ_L_ are the upper and lower wavelength bounds of a continuous band with absorption above 0.9, *t* is the thickness of the MMA. *W*_ob/*t*_ involves wavelength and the thickness of MMAs, and is a dimensionless value. Thus, it is more reasonable to use *W*_ob/*t*_ rather than *W*_RAB_ to assess the absorption efficiency of MMAs even in different frequency ranges. In our example, the MMA exhibits a *W*_ob/t_ as big as 6.01, as listed in Table 1. This value is the biggest one among all the reported MMAs including the multi-sized pyramidal MMA^28^,^33^, suggesting that the MMA proposed here is more effective in broadening the strong absorption bandwidth. This originates from our unique size regulation of the chessboard-arranged pyramids. Specifically, these regulations include three points. First, the period length (*p*) between the pyramidal units is carefully optimized to significantly enhance TM_210_ harmonics. Second, *b*_1_ is set to make a fundamental resonance corresponding to the lower frequency bound of the target absorption band (*f*_L_) and *t*_1_ to (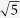
+1)*f*_L_/2. In this way, the frequency band between *f*_L_ and 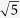
*f*_L_ is divided into 2 frequency bands and thus the fundamental and TM_210_ harmonics form two isolated strong absorption wavebands, respectively. Third, by alternately inserting small pyramidal arrays on the chessboard with *b*_2_ almost equal to *t*_1_ and *t*_2_ a little smaller than *b*_1_/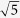, the two isolated strong absorption bands are connected to obtain a broadened continuous absorption band in a greatest efficiency. In contrast, for the previously designed pyramidal MMAs, either *b*_2_ 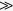 *t*_1_^33^ or *t*_2_ ≥ *b*_1_/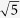
34. Moreover, *p* is seldom optimized for the deliberate enhancement of TM_210_ harmonics. Consequently, several absorption units and resonance modes contribute to only one frequency, leading to inferior absorption efficiencies. To confirm the above statements, we will demonstrate the physical implication and originality of this design strategy in the following.

**Figure 2:**
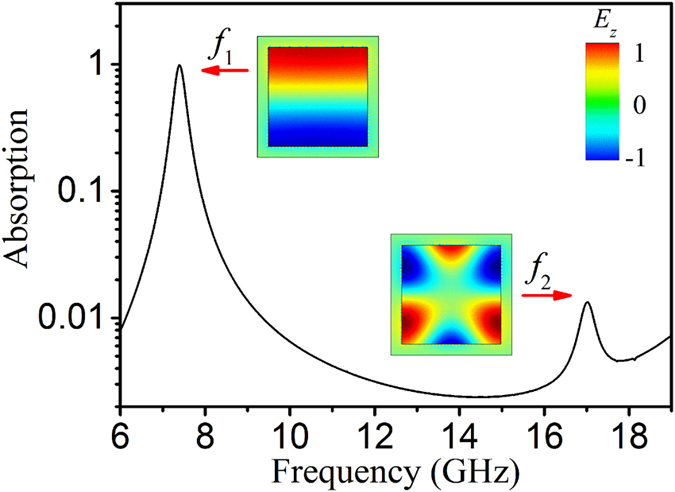
Simulated absorption spectrum of a metal-dielectric-metal resonator unit cell with *L* = 9.825 mm, which indicates that there are two resonances at frequency (*f*) at 7.4 and 17.0 GHz. The insets show the corresponding electric field distribution patterns in the unit cell at the resonance frequencies of *f*_1_ = 7.4 GHz and *f*_2_ = 17.0 GHz, respectively.

For vertically stacked multiple-layered metamaterials, the continuous broadband absorption waveband comes from the simply merged absorption peaks of the unit cells in each layer^27^,^44^. As every layer in the stack has a similar structure but different sizes, they absorb microwaves in an alike mechanism. Therefore, without loss of generality, we have selected a middle layer of the MMA with side length (*L*) of 9.825 mm to analyse the resonance behaviour for understanding the broadband absorption mechanism of the proposed MMA. Figure 2(a) indicates that there exist two absorption peaks: a strong one located at *f*_1_ = 7.4 GHz and a relatively weak one at *f*_2_ = 17.0 GHz.

When the incident waves diffract from opposite edges into a dielectric slit sandwiched by two parallel conducting metal patches, they will propagate oppositely and may excite standing waves. The resonance frequency of the standing wave can be described with equation (1)^39^.


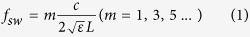


where *c*, *ε*, *L* and *m* are the speed of light in vacuum, the permittivity and length of the dielectric slit, and mode number of the harmonic, respectively. Note that only odd mode harmonics exist in this model. After substituting the parameters in the above equation with the specific dimensions of the metamaterial layer, the fundamental harmonic frequency can be calculated to be 7.36 GHz, which agrees well with *f*_1_. This indicates that the strong absorption peak results from the first order standing wave harmonic. The inset pointing to *f*_1_ reveals that the electric field distribution at *f*_1_ shows the typical characteristic of fundamental standing wave harmonics, further approving that the strong absorption peak originates from the fundamental resonance of the standing waves.

However, the absorption peak located at *f*_2_ does not fit any resonance modes predicted by Eq. (1), implying that there is a new absorption mechanism besides the fundamental harmonic resonance for the metal-dielectric-metal cavity. The *E* distribution pattern at *f*_2_ is alike a TM_210_ mode of a rectangular waveguide cavity resonator^45^,^46^. For rectangular waveguide cavity resonators, the resonance frequencies (*f*_*mnl*_) can be calculated by


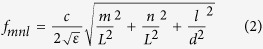


where the indices *m*, *n*, *l* represent the variation number in the standing wave patterns along the *x*, *y*, *z* directions, respectively; *L* and *d* are the resonator sizes. Equation (2) implies all the possible resonant modes along three directions. For the middle layer of the MMA with *L* = 9.825 mm, *f*_1_ corresponds to the fundamental harmonic *f*_010_ while *f*_2_ (=17.0 GHz) almost equals to 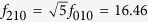 GHz (the deviation is 3.2%), suggesting that the metamaterial unit cells behave similar to rectangular waveguide cavity resonators.

**Figure 3:**
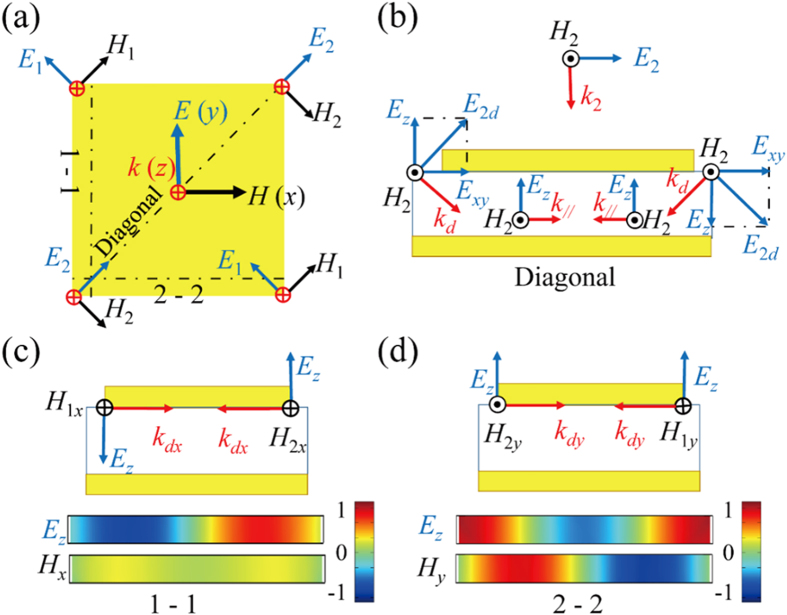
(**a**) A schematic shows the incident EM wave (*EHk*) is decomposed into two components, which diffract at four corners of the rectangular waveguide cavity resonator: for component 1 at left-top and right-bottom corners, for component 2 at right-top and left-bottom corners. (**b**) Diffracted waves from diagonal cross section. Decomposition of the diffracted wave vectors and normalized *E* field and *H* field distribution at resonance frequency of TM_210_ along (**c**) *x* direction at 1 - 1 cross section (**d**) along *y* direction at 2 - 2 cross section.

To excite TM_210_ mode, it is necessary for waves to diffract from both *x* and *y* directions. Furthermore, the diffracted waves should have an electric field (*E*) along *z* direction. Therefore, the excitation of TM_210_ resonance mode can only originate from the diffraction at the corners of the metal patches. Figure 3(a) shows the scheme of the diffractions at corners. It illustrates that the incident waves are decomposed into two component waves 1 and 2. Component 1 will diffract at the top-left and bottom-right corners, while component 2 will diffract at the top-right and bottom-left corners. As shown in Fig. 3(b), the waves diffracted from diagonal corners superpose to form standing waves, resulting in TM_210_ resonance mode. To clearly show the resonance, we decompose the diffracted waves into two components along *x* and *y* directions, and the decomposition of the wave vectors are certified by the electric field and magnetic field distribution, which clearly display the phase of the vectors. As shown in Fig. 3(c), the components along +*y* and −*y* directions are out of phase, which only enables odd mode harmonics. But the components along +*x* and −*x* directions are in phase according to the decomposition indicated in Fig. 3(d). As a result, standing waves of even harmonics are able to form. This clearly manifests that it is possible to generate TM_*mn0*_ modes, where *m* and *n* are even and odd numbers, respectively. Furthermore, it also explains well why only TM_210_ but not TM_200_ or TM_020_ exists in our design. It is the first time to discover that resonant mode TM_210_ exists in the metal-dielectric-metal unit cells. This is instructive and beneficial to extend the absorption bandwidth of their-based MMAs, though such a single unit cell only exhibits a weak intensity of the TM_210_ harmonic, as indicated by Fig. 2.

**Figure 4:**
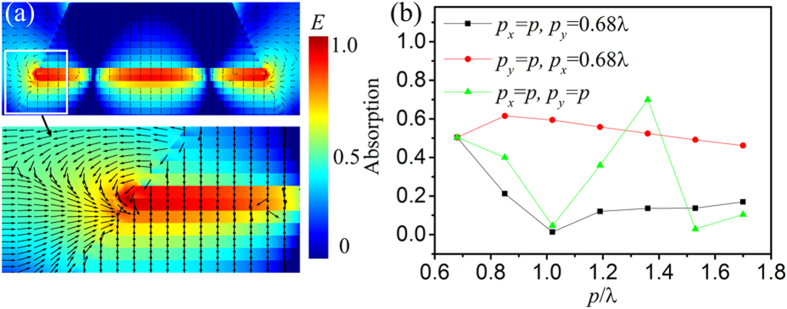
(**a**) Normalized electric field distribution with diffracted electric field vectors of the 20-layer MMA at 17 GHz along *yz* plane. (**b**) Influence of the periodic length of the MMA on the simulated absorption at 17 GHz (λ = 17.65 mm).

The absorption of TM_210_ harmonics in a metal-dielectric-metal unit cell can be significantly enhanced by vertically stacking multiple layers of unit cells. Figure 4(a) represents that for a vertically stacked multiple-layered metamaterials consisting of the big pyramids shown in Fig. 1 with *p*_*x*_ = *p*_*y*_ = 12 mm, the incident waves at the edges of metal patches are diffracted in various directions and some of the diffraction waves of neighbouring layers could access the resonance layer. Thus, the electric field intensities in the layer with TM_210_ harmonics are substantially enhanced by the diffracted electromagnetic waves of the vicinal layers. Consequently, the absorption of the MMA at 17 GHz could reach about 0.5 due to the TM_210_ harmonics (corresponding to the first left date point in Fig. 4(b)). This value is one to two orders of magnitude larger than that of the metal-dielectric-metal single layered metamaterials shown in Fig. 2.

The periodic length (*p*) between the pyramidal units has also strong influences on the electromagnetic wave diffraction property of the MMA unit cells and thus on the TM_210_ harmonic absorption. For the electromagnetic waves with *f* = 17 GHz (corresponding to λ = 17.65 mm), Fig. 4(b) indicates that when *p* along *x* direction (*p*_*x*_, *E*//*x*) increases from 0.68 to 1.7λ, the harmonic absorption at 17 GHz exhibits a minimum value of 0.01 at *p*_*x*_ = λ. As illustrated in Fig. 4(a), the absorption at 17 GHz took place in the 6^th^ layer of the pyramid with *L* = 9.825 mm, and the 1^st^ to 8^th^ layers also contribute to it. In this case, the above 12 layers work as a grating. When *p*_*x*_ = λ, the grating will reflect nearly all the incident waves according to the diffraction theory^47^, leading to a weakest absorption (see Supplementary Fig. S1 online). When *p* along *y* direction (*p*_*y*_, *E*⊥*y*) increases from 0.68 to 1.7λ, the absorption shows a maximum value of 0.62. This is because when *E* is parallel to the slit, wider slit allows more waves to permeate, enhancing TM_210_ harmonics. On the other hand, increasing *p* decreases the occupation ratio of the pyramid and thus the absorption. If the effect of the occupation ratio are excluded, increasing *p*_*x*_ and *p*_*y*_ will both increase the absorption except at *p*_*x*_ = λ (see Supplementary Fig. S2 online). There occurs a maximum absorption of 0.7 when *p*_*x*_ = *p*_*y*_ = 1.36λ. This suggests that the TM_210_ harmonic absorption could also be enhanced by the optimization of *p*.

**Figure 5:**
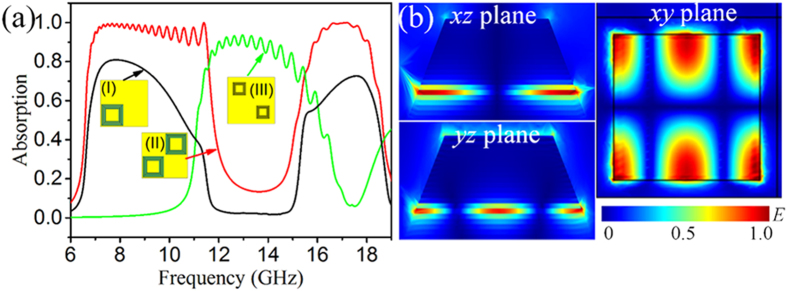
(**a**) Simulated absorption spectra of the MMAs consisting of pyramids with *p* = 1.36λ: big pyramid arrays half (I) and full filling (II) “white blanks” of a chessboard, small pyramid arrays full filling “black blanks” of a chessboard (III). (**b**) Normalized electric field distribution of structure II at 17 GHz: (left up) cross section along *xz* plane, (left down) cross section along *yz* plane and (right) central cross section of the 6^th^ layer.

Curve I in Fig. 5(a) shows the simulated absorption spectrum of the MMA based on the big pyramids shown in Fig. 1 with *p* optimized to be 24 mm. Obviously, it exhibits two absorption wavebands with the absorption within 0.4–0.8 at 7–11 GHz and 15–19 GHz. In this case, the pyramids only occupy half of the space in the “white blanks” of the chessboard and about 3/4 space in the structure is vacant. Thus, we could double the big pyramids to increase the occupancy ratio in condition that the gap between the adjacent units remains unchanged, as described by inset II of Fig. 5(a). Curve II in Fig. 5(a) indicates that when all the “white blanks” of the chessboard are filled with the big pyramids, the as-designed MMA exhibits two separated strong absorption wavebands at 7–11 GHz and 15–19 GHz due to the fundamental and TM_210_ harmonics. All the absorptions in these two wavebands are almost above 0.9.

While it is well-accepted that the strong absorption waveband at 7–11 GHz comes from the merging of the fundamental harmonics of various metal-dielectric-metal unit cells, we here pay attention to the rigorous evidence that the strong absorption waveband at 15–19 GHz is mainly attributed to the TM_210_ harmonics. According to Eq. (2), the absorption at 17 GHz may come from TM_210_ harmonic of the metal-dielectric-metal resonator with *L* = 9.825 mm, which is just the 6^th^ layer of the big pyramids (Layer 6). As shown in Fig. 5(b), the electric field (*E*) at *f* = 17 GHz is mainly located at Layer 6 as well as its adjacent layers, and it manifests a typical field distribution of TM_210_ harmonics. Similar *E* distributions are also observed at *f* = 15, 16 and 18 GHz for the layers with *L* corresponding to the frequency of TM_210_ harmonics. No other resonance modes are found except for a weak TM_030_ harmonic at 19 GHz, where the absorption is only 0.2 (see Supplementary Fig. S3 online). This fully confirms that it is the TM_210_ harmonics that dominate the absorption in 15–19 GHz. Since the MMA has a size comparable with the working wavelength, some of the incident waves may possibly scatter in various directions rather than be absorbed. To exclude this, we have calculated the absorption of the chessboard arranged the big pyramids by two different ways of calculating the volume integration of the energy loss density within the MMA and the (1 – S_11_^2^) of the MMA (see Supplementary Fig. S4 online). The two values almost equal with each other, implying that the scattering hardly occurs.

To further obtain a continuous strong absorption waveband, there is only a bandwidth of 4 GHz to compensate the absorption between them. This can be easily completed by filling the “black blanks” of the chessboard with small pyramidal arrays^33^. As a matter of fact, when the small pyramidal arrays with *L* of around 6.6 to 3.6 mm from the bottom to top are occupied in the “black blanks” of the chessboard, they exhibit a strong absorption in 11–15 GHz as indicated by curve III in Fig. 5(a). Therefore, after simultaneously arranging the two kind of pyramidal unit cells in the “white blanks” and “black blanks” of the chessboard, the fulfilled metamaterial can display a strong absorption of above 0.9 in the whole frequency range of 7–18 GHz. This is verified both in simulation and experiment as shown in Fig. 1(b).

Above all, the broad absorption bandwidth comes from three sources: the fundamental harmonics of the big and small pyramids, as well as the TM_210_ harmonics of the big pyramids. The as-designed broadband pyramidal MMAs should make full use of TM_210_ harmonics of the big pyramids at the high frequency absorption by regulating *p* to ensure the excellent absorption efficiency. In addition, the sizes of the big and small pyramids should approximately obey the following equations: 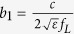 to follow Eq. (1); 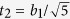 to ensure the connection of the absorption bands provided by the fundamental harmonics of the small pyramids and the TM_210_ harmonics of the big pyramids; 
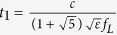
 to make the big and small pyramids contribute to half of the frequency band from 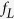 to 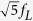, respectively; 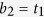 to ensure the connection of the absorption bands provided by fundamental harmonics of the big and small pyramids. Under the above conditions, the size parameters should be made some further optimization to achieve a continuous strong absorption waveband. In our example, we obtain a strong absorption bandwidth of 11 GHz from 7 to 18 GHz. In contrast, if ignoring the contribution from TM_210_ harmonics, the absorption waveband only covers the frequency range of 7–15 GHz, which is around 8 GHz in width. This means that the bandwidth is increased by 38% due to TM_210_ harmonics of the big pyramids. Compared with the traditional simple design which uses only the fundamental harmonic absorption in 7–11 GHz and 11–15 GHz from the big and small pyramids respectively, our MMA designed here has a tripled bandwidth. According to Eq. (2), there are higher order harmonics at higher frequencies for the metal-dielectric-metal resonance cavity unit cells. Therefore, some isolated strong absorption wavebands are expected at frequencies exceeding 19 GHz^44^, where we do not have a focus here.

**Figure 6:**
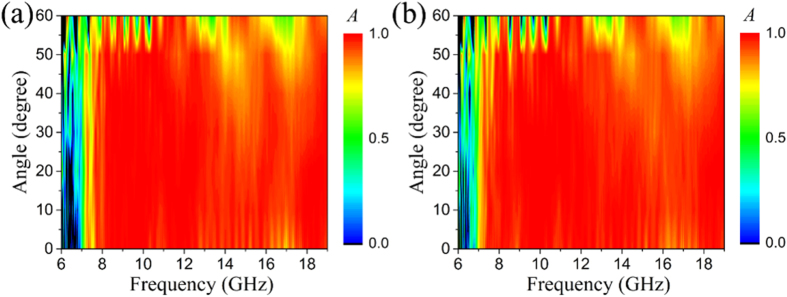
Measured absorption of the proposed MMA under illumination of (**a**) TE and (**b**) TM waves with incident angles from 0 ° to 60 ° in the frequency range of 6–19 GHz.

The measured absorption contour map for TE and TM polarization with incident angle versus frequency is plotted in Fig. 6. When the incident angle is smaller than 50°, the MMA maintains its absorption of above 0.9 in the frequency range of 8–19 GHz, manifesting the excellent angular independence of absorption. Comparison of Fig. 6(a) with (b) indicates that the MMA presents polarization insensitivity even at an incident angle of 60 °. The angular and polarization insensitivity of the MMA are beneficial for many applications such as stealth, electromagnetic compatibility and solar energy collection^1^,^8^,^19^. The as-proposed design method is expected to be flexible as it can be extended to other frequency ranges due to the form-invariance of Maxwell equations.

## Conclusion

In conclusion, commonly used metamaterial unit cells of square-shaped metal-dielectric-metal cavities behave like rectangular waveguide cavity resonators. By the wave diffraction at diagonal corners they may excite TM_210_ harmonics, which can be further enhanced by multi-layer overlapping and regulation of period length between adjacent unit cells. On such a basis, a design method to achieve broadband metamaterial absorbers is proposed by employing both fundamental and TM_210_ harmonics from the unit cells of metal-dielectric-metal cavities. A broadband metamaterial absorber (MMA) is accordingly designed and experimentally verified to show an absorption of above 0.9 in the frequency range of 7–18 GHz, whose bandwidth is tripled with respect to the original simple configuration which only uses fundamental harmonics. The as-proposed design strategy provides a general route to greatly extend the absorption bandwidth of MMAs without increasing the total thickness.

## Methods

The absorptions and field distributions of the MMAs are numerically simulated and calculated using a Finite-Element-Method based software package Comsol Multiphysics. The MMA is fabricated by a computer assisted mechanical engraving machine. First, 20 layers of 200 × 200 mm printed circuit boards (PCBs), each of which consists of a copper film with a thickness of 0.018 mm covered on a 0.15-mm-thick FR4 board, are bounded together to form a stack using an adhesive with a thickness of 0.05 mm and almost the same dielectric constant as FR4. A copper film is added to the bottom of the stack to suppress the transmission. Afterwards, the above PCB stack is subjected to mechanical engraving, a 200 × 200 mm sample containing 49 units is obtained as shown in Fig. 1(a). The reflection (*R*) of the MMA is measured in a microwave anechoic chamber using an Agilent Network Analyzer N5230A.

## Additional Information

**How to cite this article**: Long, C. *et al*. Broadening the absorption bandwidth of metamaterial absorbers by transverse magnetic harmonics of 210 mode. *Sci. Rep*. **6**, 21431; doi: 10.1038/srep21431 (2016).

## Supplementary Material

Supplementary Information

## Figures and Tables

**Table 1:** Comparison of absorption properties of the planar MMAs reported in literatures and this work proposed here.

Feature of MMAs	Absorption waveband	Thickness	*W*_ob/*t*_	Reference
Truncated pyramidal	7.8–14.7 GHz	5 mm	3.61	27
Double-corrugated structure	7.8–16 GHz	5 mm	3.94	28
1-layer donut-shape	5.03–5.13 GHz	0.8 mm	1.45	41
3-layer crosses structure	4.45–4.95 THz	3.8 μm	1.79	17
Double-layered grating	0.25–2.5 THz	620 μm	1.74	45
Saw-toothed multi-layer slab	0.3–4.7 μm	1 μm	4.40	10
Multiple-layer pyramidal	1–14 μm	3 μm	4.33	30
Hyperbolic waveguide taper array	2.9–5 μm	40 μm	5.00	33
Multi-sized sow-wave hyperbolic	0.5–2.5 μm	56 μm	3.75	34
Truncated pyramidal	0.2–5.8 μm	56 μm	1.00	35
1-layer mixed-size square hole	583–613 nm	15 μm	0.20	14
Multi-sized Pyramidal using TM_210_	7–18 GHz	4.36 mm	6.01	This work
