# Supplementary material for: Broadening the absorption bandwidth of metamaterial absorbers by transverse magnetic harmonics of 210 mode
Source: Sci Rep. 2016 Feb 18;6:21431. doi: 10.1038/srep21431 (PMC4757821; doi:10.1038/srep21431)
Supplement: Supplementary Information [file srep21431-s1.doc]

Supplementary Information

**Broadening the absorption bandwidth of metamaterial absorbers by transverse magnetic harmonics of 210 mode**

**Chang Long,^1, +^ Sheng Yin,^1, +^ Wei Wang,^1^ Wei Li,^1,*^ Jianfei Zhu,^2^ and Jianguo Guan^1,*^**

^1^State Key Laboratory of Advanced Technology for Materials Synthesis and Processing, Wuhan University of Technology, Wuhan, 430070, China

^2^State Key Laboratory for Modern Optical Instrumentation, Centre for Optical and Electromagnetic Research, College of Optical Science and Engineering, Zhejiang University, Hangzhou 310058, China

^*^ Corresponding authors: [wellee@whut.edu.cn](mailto:wellee@whut.edu.cn) and [guanjg@whut.edu.cn](mailto:guanjg@whut.edu.cn)

+ These two authors contributed equally to this work


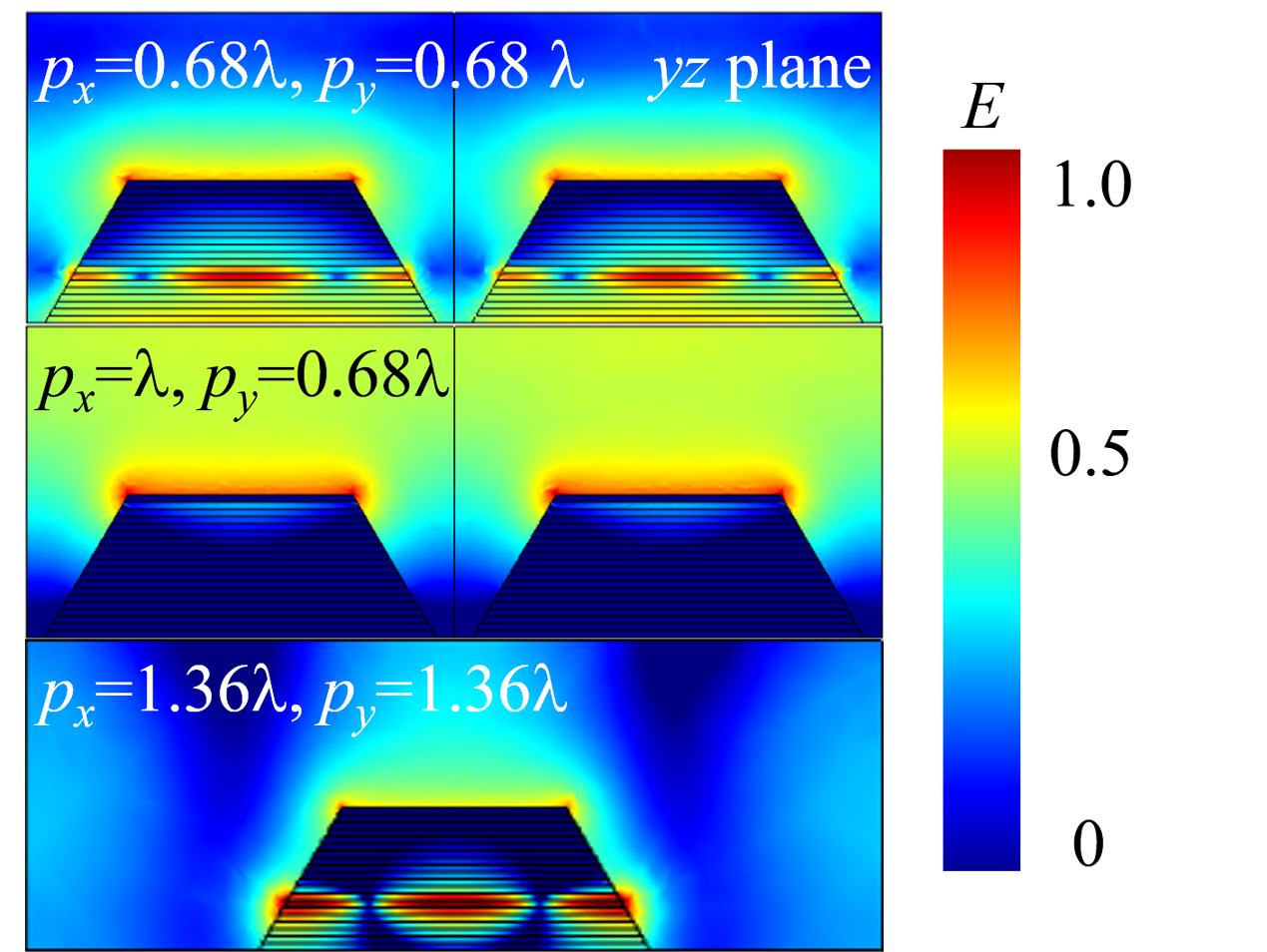


Fig. S1. Normalized *E* field distribution of the MMAs at 17 GHz with different periodic length along *yz* plane.


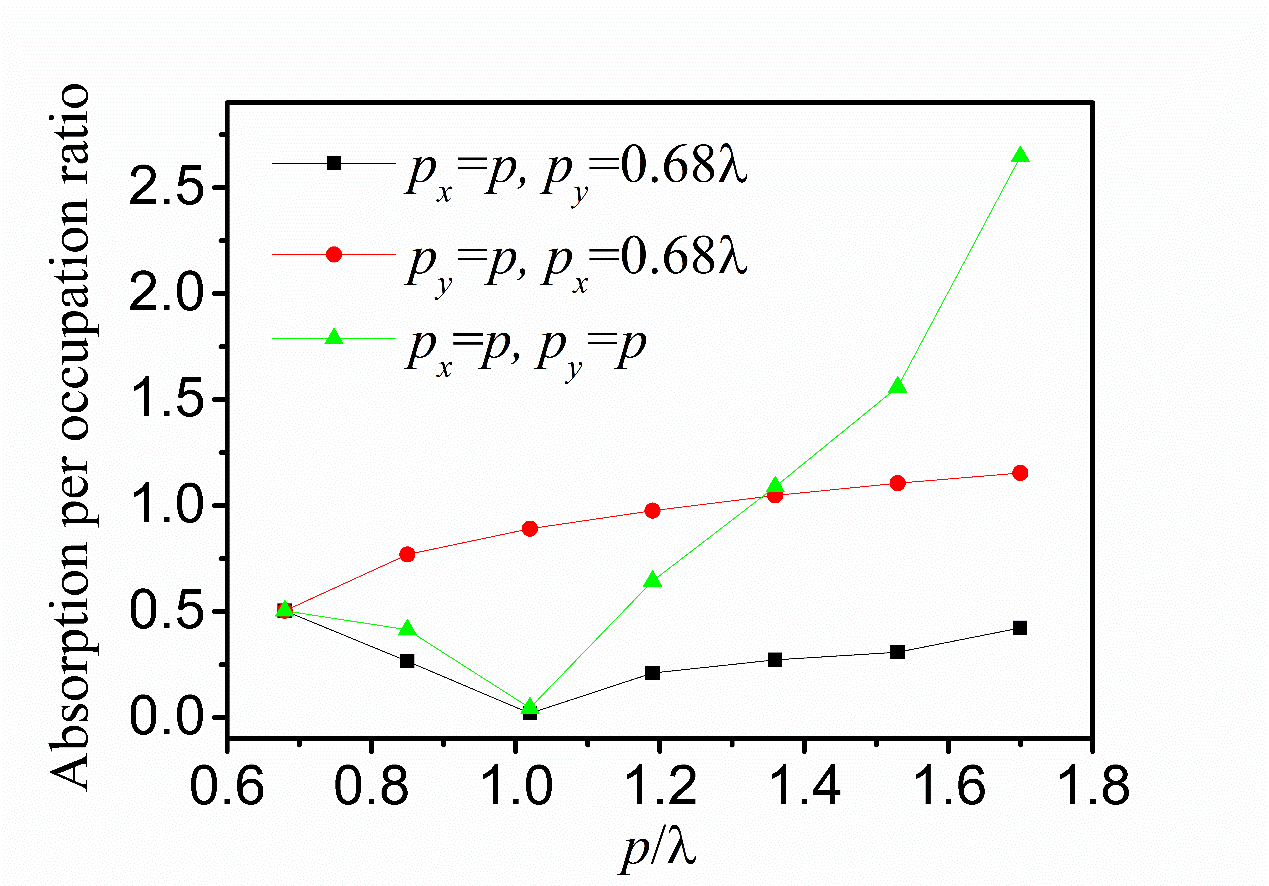


Fig. S2. Influence of the periodic length of metamaterial unit cells on the absorption (*A*) per occupation ratio of the MMAs at 17 GHz (λ=17.65 mm). The absorption per occupation ratio is defined as *A*/(12^2^/*p_x_p_y_*).


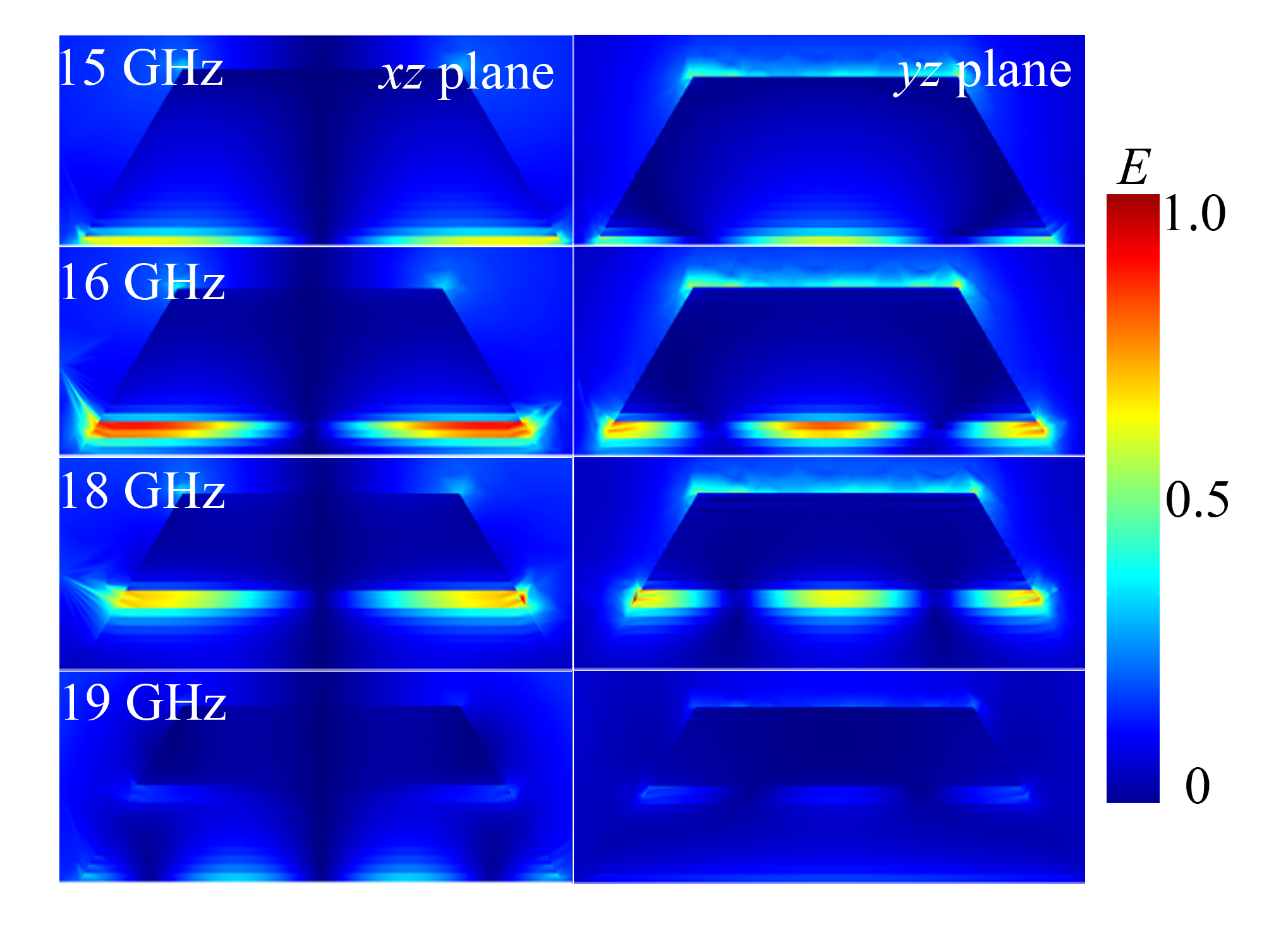


Fig. S3. Normalized *E* field distribution of the big pyramids arranged in chessboard manner at *xz* and *yz* plane for electromagnetic waves with different frequencies.


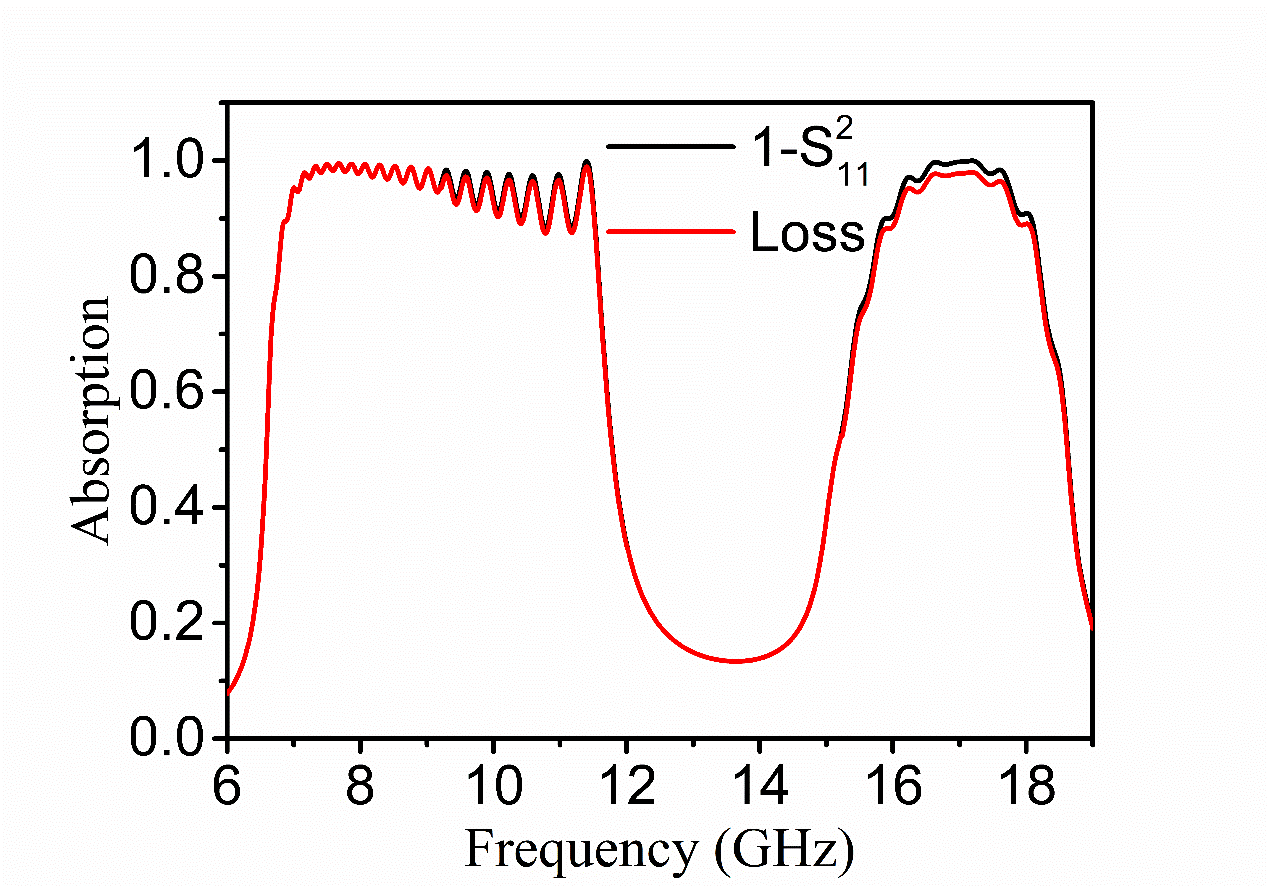


Fig. S4. Absorption performance of the big pyramids arranged in chessboard manner. The absorption properties are calculated by two ways: the black line is calculated by 1 – S_11_^2^ and the red line is calculated by volume integral of energy loss density within the MMA.
